# *Rmax*: A systematic approach to evaluate instrument sort performance using center stream catch^☆^

**DOI:** 10.1016/j.ymeth.2015.02.017

**Published:** 2015-07-01

**Authors:** Andrew Riddell, Rui Gardner, Alexis Perez-Gonzalez, Telma Lopes, Lola Martinez

**Affiliations:** a Flow Cytometry Core Facility, EMBL-Heidelberg, Germany; b Flow Cytometry Facility, Wellcome Trust-Medical Research Council, Cambridge Stem Cell Institute, United Kingdom; c Cell Imaging Unit, Instituto Gulbenkian de Ciência, Oeiras, Portugal; d Single Cell Facility, ETH Zurich, Basel, Switzerland; e Flow Cytometry Unit, Spanish National Cancer Research Center (CNIO), Madrid, Spain

**Keywords:** Flow cytometry, *Rmax*, Recovery, Purity, Yield, Drop-charge delay, Sort, Instrument performance, Quality control

## Abstract

Sorting performance can be evaluated with regard to Purity, Yield and/or Recovery of the sorted fraction. Purity is a check on the quality of the sample and the sort decisions made by the instrument. Recovery and Yield definitions vary with some authors regarding both as how efficient the instrument is at sorting the target particles from the original sample, others distinguishing Recovery from Yield, where the former is used to describe the accuracy of the instrument’s sort count. Yield and Recovery are often neglected, mostly due to difficulties in their measurement. Purity of the sort product is often cited alone but is not sufficient to evaluate sorting performance. All of these three performance metrics require re-sampling of the sorted fraction. But, unlike Purity, calculating Yield and/or Recovery calls for the absolute counting of particles in the sorted fraction, which may not be feasible, particularly when dealing with rare populations and precious samples. In addition, the counting process itself involves large errors.

Here we describe a new metric for evaluating instrument sort Recovery, defined as the number of particles sorted relative to the number of original particles to be sorted. This calculation requires only measuring the ratios of target and non-target populations in the original pre-sort sample and in the waste stream or center stream catch (CSC), avoiding re-sampling the sorted fraction and absolute counting. We called this new metric *Rmax*, since it corresponds to the maximum expected Recovery for a particular set of instrument parameters. *Rmax* is ideal to evaluate and troubleshoot the optimum drop-charge delay of the sorter, or any instrument related failures that will affect sort performance. It can be used as a daily quality control check but can be particularly useful to assess instrument performance before single-cell sorting experiments. Because we do not perturb the sort fraction we can calculate *Rmax* during the sort process, being especially valuable to check instrument performance during rare population sorts.

## Highlights

• Recovery is a global sort performance parameter.
• Standard Recovery calculations are error prone.
• *Rmax* provides accurate and precise assessment of Recovery.
• *Rmax* is quick to perform and can be universally applied.

## 1 Introduction

### 1.1 An overview of the sort process

In jet-in-air sorters, a fine jet of liquid is expelled from a nozzle coupled to a piezo crystal. An electrical sine wave is imposed on the piezo crystal causing a uniform sonic wave to be imprinted onto the jet, which under strict conditions, will break into droplets with a defined frequency at a specific location [1]. Particles for analysis and sorting are injected into the core of the nozzle and confined to a single file through hydrodynamic forces. Once in the jet’s core, particles will ideally move one at a time through a focused laser beam spot, allowing light scatter and fluorescent measurements to be taken from each particle. Classification of particles can be made, and because the break-off point (BOP) of the jet is stable in time, drops containing desired target particles can be selectively charged, deflected by an electrostatic field, and finally collected in a container. In reality, mono-dispersed particles will follow a Poisson probability profile with a proportion of particles in a singlet, doublet, triplet, etc. spacing. Coincidence events occur when two or more particles arrive together at the laser interrogation point or within the boundaries of a drop. Electronics are designed to handle these coincidence events. Hardware coincidence events, which are detectable coincidences that happen within the duty cycle (dead-time) of the electronics lead to the measurement being aborted. Drop coincidence events are controlled differently according to the selected sort mode. For instance, in a Beckman Coulter MoFlo cell sorter, a *Single-cell* mode favors a precise count in the deflection of highly pure target particles, whereas a *Purify* mode favors a high Purity without count precision. *Enrich* mode prioritize a high Recovery of the target particle, producing sort fractions with compromised Purity. Ideally, the probability of a particle being sorted depends on the relative frequencies of target and non-target events for each of the coincidences events and should follow the Poisson and Binomial distributions. The outcome of a sort in terms of Recovery (*Single-cell* and *Purify* modes) and Purity (*Enrich* mode) can be anticipated this way as a function of the average event rate, the droplet generation frequency and the original frequency of the target population.

### 1.2 Issues in standard evaluation of performance of a jet-in-air sorter

As simple as this description may be, jet-in-air sorting is a highly complex process due to the inherent requirements for high fluidic stability, electronic and timing precision. Making sure the instrument is well calibrated and setup for sort requires the rigorous assessment of its performance. Several quality control metrics can be considered when evaluating the performance and outcome of a given sort, among them Purity and Recovery are two of the most frequently used. Purity, referring to the percentage of target particles out of the total in the sort fraction, can be readily assessed with the analysis of the sort product. As for Recovery, there is some degree of confusion surrounding its definition as this term has been used to describe two separate sort performance metrics. Recovery has been defined as the number of target particles collected in the sorted fraction divided by the number of sort decisions indicated by the instrument’s sort counters [2], and has also been defined as equivalent to and as a substitute for Yield, i.e., the fraction of the number of target particles collected by sorting, relative to their original numbers in the pre-sort sample [3]. In the former definition, calculating Recovery requires the absolute counting of target particles in the sorted sample, whereas in the latter definition, absolute counting of target particles in both the original and sorted fractions is required. Recovery succeeds in situations in which Purity readings fail to do so. One of the most noticeable instances of this involves the incorrect determination of the instrument drop-charge delay, commonly referred to simply as drop delay. After a target population of interest has been identified, their isolation in an electrostatic jet-in-air sorter requires a precise knowledge of the time delay of the deflection circuitry to the arrival time of the target particle at the BOP. While sorting under normal conditions, when the event rate does not exceed more than one fifth of the droplet formation frequency, the effect of an incorrect drop-charge delay estimation will most likely be to sort an empty drop, and the particle of interest will be lost through the waste stream, leading to a reduction of target particles in the collection tube. Consequently, Purity will hardly be affected unlike Recovery, which will be highly compromised. At higher event rates, i.e., at higher average drop occupancies, Purity will begin to be affected, and for every non-target particle sorted, a target particle will still be lost. From this, it is clear that errors in the timing of the deflection circuitry will affect Recovery more than Purity, making the former the most suitable candidate when evaluating a sorter’s performance.

### 1.3 Recovery in drop-charge delay determination

The concept of Recovery is already incorporated in different drop-charge delay determination methods, which are indirectly used to evaluate instrument performance. Drop-charge delay values can be calculated by several methods depending on the instrument and the manufacturer. Although the time delay of the deflection circuitry can be roughly estimated by calculation, a precise definition requires the empirical evaluation of the Recovery of target particles while sorting over a given subset of contiguous time delay values. This is the basis of the slide-based delay methods [2,4] and recently, the development of automated systems such as the Accudrop™ [5–7] and related systems [8,9]. Confirming the accuracy of the delay values currently requires the assessment of the instrument recoveries according to classical approaches that consist in repeated sampling and absolute counting of target particles in the original sample and the sorted fraction at the end of the sort. The process of counting particles in a given volume has a large amount of error deriving from concentration and volume estimations [10,11]. Although methods based on the Coulter principle [12,13] or cytometric counting beads [14] offer better accuracy due to the larger number of particles being sampled, they may still suffer from volume errors as well. In addition, all of these methods when applied to the calculation of Recovery will require sampling of the original pre-sort sample and the sort product, which might not be feasible when dealing with precious samples and sorting of rare populations.

### 1.4 A new measure of Recovery: *Rmax*

In order to identify the contribution of several factors affecting sort performance and to evaluate strategies to circumvent these issues, a reliable and fast method to assess instrument sort performance is imperative. In this article, we describe a new ratio-metric method to evaluate instrument sort Recovery. Maximum Recovery or *Rmax* bypasses the need of absolute counting, requiring only the cytometric measurement of target to non-target ratios in the pre-sort sample, the sorted fraction, and the waste stream or the center stream catch (CSC). Its mathematical expression can be further simplified in high Purity sorts where the purity of the sorted fraction approaches 100%. *Rmax* then becomes the ratio of target to non-target particles in the original presort sample and the CSC, avoiding the need to analyze the sorted fraction to measure *Rmax*. Additionally, the method allows the assessment of instrument Recovery at any given time during a sort, rather than after completion. The results support the use of *Rmax* as a quality control (QC) metric to monitor and troubleshoot factors affecting cell sorter performance before and during a sort, and to evaluate the accuracy of drop-charge delay methods.

## 2 Materials and methods

### 2.1 Reagents

Flow-Check™ Fluorospheres (Beckman Coulter, Inc. Miami USA) were used during Beckman Coulter MoFlo cell sorter setups in order to maximize the instrument’s optical alignment and also during the identification of the instrument drop-charge delays. SPHERO™ Drop Delay Calibration Particles (Spherotech, Inc. Illinois USA) were used to set the drop-charge delays in the FACSAria cell sorter. Blank, FITC^+^, PE^+^ and APC^+^ BD CaliBRITE™ beads (BD Biosciences San Jose CA USA) diluted into 1 ml of PBS buffer supplemented with 2% BSA (Sigma–Aldrich St Louis MO USA) were used during the sorting assays. CountBright™ absolute counting beads (Molecular Probes® Eugene OR USA) were used in the absolute counting of sort products during calculations of traditional sort recoveries.

### 2.2 Instrument setup

Beckman Coulter MoFlo sorters were equipped with a 488 nm laser (Innova™ 90C Argon, Coherent Inc.) set between 80 and 100 mW at the laser intersection point. In some experiments the instrument was configured with a 100 μm nozzle at 210 kPa (30 psi) and 43 kHz droplet generation. In the evaluation of the drop-charge delay and *Rmax* validation, an additional instrument setup with a 70 μm nozzle at 414 kPa (60 psi) and 95 kHz droplet generation was used. Both setups were performed using a 1-drop deflection sort mode to give the minimal condition of coincidence events [15,16]. Beckman Coulter Summit software was used to control the instrument.

BD FACSAria flow cytometers (BD Biosciences, San Jose, CA) was equipped with a 488 nm (15–20 mW output) Coherent Sapphire solid-state laser. In the evaluation of the drop-charge delay and in *Rmax* determination the instrument was configured with a 70 μm nozzle, sheath pressure of 483 kpa (70 psi) and 90 kHz droplet generation. All sorts were carried out with a 0-16-0 sort precision mask, unless stated otherwise. BD FACSDiva software was used to control the instrument.

### 2.3 Setting the drop-charge delay

FACSAria drop-charge delay configuration procedure was performed as described in the operators manual (FACSAria I, FACSAria II and FACSAria III). The automated method of drop delay determination was also performed in the FACSAria II and FACSAria III as described [7]. The MoFlo’s drop-charge delay was evaluated following the method described in the manual [2] and additionally by a homemade Calibrator device adapted to the MoFlo [17,18]. There were two methods used to determine the drop-charge delay for the Calibrator. The first method was to determine the minimum bead flash in the waste stream with the maximum bead flash in the sort stream. The second method modified the first method by including careful alignment, focusing of the laser and camera in the Calibrator. Flow Check™ beads were run at 2000 events per second (e.p.s.) to ensure approximately 100% sort efficiency. The laser was set to 80 mW of power. The sort decision was made on a FSC-Area vs. SSC-Area plot with a sort region on an empty part of the plot. The sort region was configured to give a NOT sort decision, consequently sorting all triggered events. In order to find the correct drop-charge delay, the MoFlo console Delay parameter was adjusted to maximize the intensity of the deflected stream and minimize the intensity of the waste stream. The drop-charge delay was identified as the one giving the minimum fluorescence intensity in the waste stream.

### 2.4 Capturing the center stream catch for *Rmax* calculation

During sorting, the center stream particles were collected by either placing a 5 ml Polypropylene tube (BD Falcon™ 352063) containing 500 μl of PBS BSA 2% directly under the center stream above the waste collection (FACSAria) or by adjusting the center stream charge setting in Summit software in order to deflect it into a CSC collection tube placed opposite to the sort deflection (MoFlo). Around 5 ml of CSC were collected. Before the analysis, the CSC tubes were centrifuged in order to speed-up the acquisition of the CSC beads. After centrifugation at 300 g for 2 min, most of the supernatant was discarded by careful pipetting leaving approximately 300 μl of the bottom CSC volume. CSC particles were suspended by vortexing and analyzed on a cytometer together with the original pre-sort and sorted fractions. The number and frequencies of target and non-target events in different fractions were recorded for *Rmax* calculations and purity assessment.

### 2.5 Investigating the homemade MoFlo Calibrator device

To investigate the accuracy of the drop-charge delay determination of the Calibrator device, two of the authors APG and AR set up a trial on the MoFlo. APG would randomize the drop-charge delay on the CSU console of the MoFlo and cover the readout. AR would then determine the optimum drop-charge delay using the Calibrator by the modified procedure. This was repeated 10 times. Verification of the MoFlo Calibrator drop-charge delay was performed by Summit software’s Drop Delay Test coarse method and *Rmax* calculated by scanning through +3 and −3 1/16ths of the drop around the optimum setting suggested by the first two methods.

### 2.6 Sort Recovery calculation based on Poisson–Binomial theory

A MoFlo cell sorter equipped with a 70 μm nozzle 414 kPa (60 psi) sheath pressure and 96.43 kHz droplet generation frequency was configured for replica *Single 1-drop* mode sorts. A total of 10^5^ target FITC^+^ particles were isolated out of a FITC-PE CaliBRITE™ bead mix, sorted at around 5000 e.p.s. At the end of each sort, Summit software reports on the average event rate and the percentage of FITC^+^ particles out of the total triggering events were collected; together with the number of drop abort events reported by the MoFlo CSU console. The number of hard aborts was estimated by Poisson–Binomial (P–B) theory [19,20]. The expected Recovery $R_{\text{P}\text{"–"}\text{B}}$ associated with each replica sort was calculated based on the number of sort decisions, *Sd*, and the number of original target particles lost to drop aborts $A_{t(D)}$ and hard aborts $A_{t(H)}$

$$R_{\text{P}\text{"–"}\text{B}}=\frac{\mathrm{Sd}}{\mathrm{Sd}+A_{t(D)}+A_{t(H)}}$$

### 2.7 Sort Recovery calculations based on absolute counting of the sorted target particles

In experiments to validate Recovery by *Rmax*, target FITC^+^ CaliBRITE™ beads (10^5^ in total) were sorted under *Single 1-drop* mode at average rates ≈5000 e.p.s. in a MoFlo configured with a 70 μm nozzle (414 kPa sheath pressure and around 95 kHz droplet generation frequency). Sorted particles were collected into Eppendorf™ tubes containing 100 μl of PBS BSA 2%. The CSC was collected and processed as above. The absolute count of sorted target particles, *S_t_*, was calculated by multiplying sort target concentrations by the volume of the sorted fraction. *S_t_* concentration was calculated with CountBright™ beads and with an automated slide counter (Bio-Rad TC10 system) following manufacturer’s instructions. The absolute volume of each sorted faction was calculated immediately after sort. Briefly, the empty weight (*W_E_*) of each sort collection tube was recorded before the addition of 100 μl of sort collection media and upon sort completion (*W_S_*). After removal of 50 μl from gently suspended *S_t_* fractions (used in bead-based concentration measurement), the remaining collection tube weight (*W*_Δ_) was measured. The total volume of the sort fraction (*V_St_*) was then calculated as:

$$V_{\mathrm{St}}=0.05\;\text{ml}\times \left[\frac{W_{S}-W_{E}}{W_{S}-W_{\Delta }}\right]$$

Recovery associated with each replica sort was calculated based on the number of sorted target particles derived from each counting method (*S_t_*) and the number of original target particles ran by the instruments upon completion of the sort, estimated from Poisson–Binomial theory:

$$R=\frac{S_{t}}{\mathrm{Sd}+A_{t(D)}+A_{t(H)}}$$

## 3 Results

### 3.1 *Rmax*: a measure of instrument maximum sort Recovery

Evaluating the source of cell loss during a sort experiment is generally difficult using the traditional methods to measure Recovery. Even when comparing the number of sort decisions made by the instrument with the final number of cells in the sorted tube, it remains very hard to ascertain whether loss occurred due to instrument failure or simply cell death and/or cell adherence to the tube walls. To evaluate cell loss due to failure or non-optimal calibration of the instrument we derived a surrogate expression of Recovery that relates the number of sort decisions with the number of sorted cells lost in the waste stream, and is independent of absolute counts or sorting the whole sample. First, two populations must be defined. The target population (*t*), consisting of all particles within the sort gate and the non-target population (*nt*), comprised by the remaining triggering particles. When acquiring and sorting target particles of a fraction *α* of the original sample, it is fair to assume that both the target and non-target particles will have been either deflected into the sorted tube or lost in the waste through the center stream. Mathematically, this can be translated into the following: when a fraction *α* of the original sample is sorted, the absolute number of original target particles present in this fraction *α* (*αO_t_)* equals the sum of target particles in the sort collection (*S_t_*) and center stream (*C_t_*) compartments:

(1) $$\alpha O_{t}=S_{t}+C_{t}$$

The same relationship can be assumed also for non-target (*nt*) particles,

(2) $$\alpha O_{\mathrm{nt}}=S_{\mathrm{nt}}+C_{\mathrm{nt}}$$

Sort Recovery, defined as the fraction of particles of interest collected by sorting relative to their original number in the pre-sort sample can be expressed, when applied to the sort of fraction *α*, as:

(3) $$R=\frac{S_{t}}{\alpha O_{t}}\;\text{with}\;0<\alpha <1$$

From Eq. (1), an alternative expression for Recovery as a function of target particle loss to the CSC compartment can be derived:

(4) $$\frac{S_{t}}{\alpha O_{t}}=1-\frac{C_{t}}{\alpha O_{t}}$$

Or

(5) $$\mathrm{Rmax}=1-\frac{C_{t}}{\alpha O_{t}}$$

Essentially, sort Recovery can be defined as a function of the number of target particles in the CSC relative to the number of original target particles contained within a sorted fraction *α* of the original sample. Assuming stability over time in both the instrument operation and the sample properties, Recovery as expressed in Eq. (4) or Eq. (5) can be regarded as the maximum Recovery that can be expected for a particular instrument under a defined set of conditions such as nozzle size, frequency of drop formation, particle speed and applied sort mode. We termed this metric *Rmax* or maximum sort Recovery. Solving Eq. (2) for *α* and replacing its value in Eq. (5) it is possible to derive a simple relationship between *Rmax* and the absolute number of target and non-target particles in each compartment:

(6) $$\mathrm{Rmax}=1-\frac{O_{\mathrm{nt}}}{O_{t}}\cdot \frac{C_{t}}{C_{\mathrm{nt}}+S_{\mathrm{nt}}}$$

Assuming the ratio of non-target to target particles in each compartment remains constant throughout the sorting process, i.e. independent of any given sorted fraction *α*, *O_nt_*/*O_t_* ratio in Eq. (6) will be equivalent to that observed at the interrogation point while sorting a fraction *α* of the original sample. However, determining *Rmax* with Eq. (6) still requires the knowledge of the absolute number of target and non-target particles in the sorted tube and CSC.

#### 3.1.1 *Rmax* expressed in ratios

To circumvent these limitations, *Rmax* can be expressed as a function of the ratios of target and non-target particles in these compartments. Defining these ratios as:

(7.1) $$O^{*}=\frac{O_{\mathrm{nt}}}{O_{t}}\text{,}\;\text{for the original pre-sort sample}$$

(7.2) $$S^{*}=\frac{S_{\mathrm{nt}}}{S_{t}}\text{,}\;\text{for the sorted fraction}$$

(7.3) $$C^{*}=\frac{C_{\mathrm{nt}}}{C_{t}}\text{,}\;\text{for the CSC}$$

Replacing these expressions in Eq. (5), we obtain:

(8) $$\mathrm{Rmax}=1-O^{*}\cdot \frac{C_{t}}{S^{*}S_{t}+C^{*}C_{t}}$$

To remove the explicit contribution of *S_t_* and *C_t_*, a new relation needs to be introduced. An equation relating the number of target and non-target particles in all compartments can be obtained by dividing Eq. (2) by Eq. (1):

(9) $$\frac{O_{\mathrm{nt}}}{O_{t}}=\frac{S_{\mathrm{nt}}+C_{\mathrm{nt}}}{S_{t}+C_{t}}\text{,}$$

Which, by using Eqs. (7.1)–(7.3), can also be rewritten as:

(10) $$O^{*}=\frac{S^{*}S_{t}+C^{*}C_{t}}{S_{t}+C_{t}}$$

Solving Eq. (10) for *S_t_* and replacing its value in Eq. (8), we obtain after rearrangement and simplification a complete expression for *Rmax*:

$$\mathrm{Rmax}=\frac{C^{*}-O^{*}}{C^{*}-S^{*}}$$

Or more explicitly:

(11) $$\mathrm{Rmax}=\frac{\frac{C_{\mathrm{nt}}}{C_{t}}-\frac{O_{\mathrm{nt}}}{O_{t}}}{\frac{C_{\mathrm{nt}}}{C_{t}}-\frac{S_{\mathrm{nt}}}{S_{t}}}$$

Eq. (11) provides a simple relationship for *Rmax* exclusively dependent on ratios of target and non-target particles in each compartment and can be readily applied in the calculation of sort Recovery at any given time during a sort by cytometric inspection of the original sample, the sorted fraction and the collected CSC.

#### 3.1.2 *Rmax* expressed in percentages

*Rmax* can also be described in terms of percentages of target and non-target particles. Since the ratio of target and non-target particle numbers in each compartment is equivalent to the ratio of their percentages, a new expression of *Rmax* in terms of percentages can be easily derived:

(12) $$\mathrm{Rmax}=\frac{\frac{\%C_{\mathrm{nt}}}{\%C_{t}}-\frac{\%O_{\mathrm{nt}}}{\%O_{t}}}{\frac{\%C_{\mathrm{nt}}}{\%C_{t}}-\frac{\%S_{\mathrm{nt}}}{\%S_{t}}}$$

#### 3.1.3 Simplified *Rmax* equations for high Purity and rare target particle sorts

As the Purity of a sort reaches values close to maximum, the ratio of non-target to target particles in the sorted fraction becomes negligible, i.e. $\frac{S_{\mathrm{nt}}}{S_{t}}\approx 0$. Under these conditions of high Purity, the expression for *Rmax* simplifies to:

(13) $${\mathrm{Rmax}}_{P\to 1}=1-\frac{O_{\mathrm{nt}}}{O_{t}}\cdot \frac{C_{t}}{C_{\mathrm{nt}}}$$

Therefore, when applied to high Purity sorts (*Single* and *Purify* sort modes in a MoFlo cell sorter, for instance), *Rmax* calculation from Eq. (13) will only require measuring the ratios of target to non-target particles at the interrogation point and at the CSC after cytometric re-sampling.

Considering again *Rmax* as a function of target and non-target percentages, since the values of $\%S_{\mathrm{nt}}$ are also negligible as sort Purity approaches maximum, the expression in percentages for *Rmax* in Eq. (12) under these conditions becomes a function of original and CSC target percentages exclusively:

(14) $${\mathrm{Rmax}}_{P\to 1}=1-\frac{\%C_{t}}{\%O_{t}}\cdot \frac{100-\%O_{t}}{100-\%C_{t}}$$

A further simplification of *Rmax* is achieved when dealing with high Purity sort modes of rare original target populations (i.e. when $\%O_{t}\to 0$). Since both %*O_t_* and %*C_t_* become negligible, the ratio $\frac{100-\%O_{t}}{100-\%C_{t}}\approx 1$ Eq. (14) and the expression for *Rmax* becomes:

(15) $${\mathrm{Rmax}}_{P\to 1\text{,}\%O_{t}\to 0}=1-\frac{\%C_{t}}{\%O_{t}}$$

### 3.2 *Rmax* as a method to evaluate and troubleshoot instrument performance

**Fig. 1:**
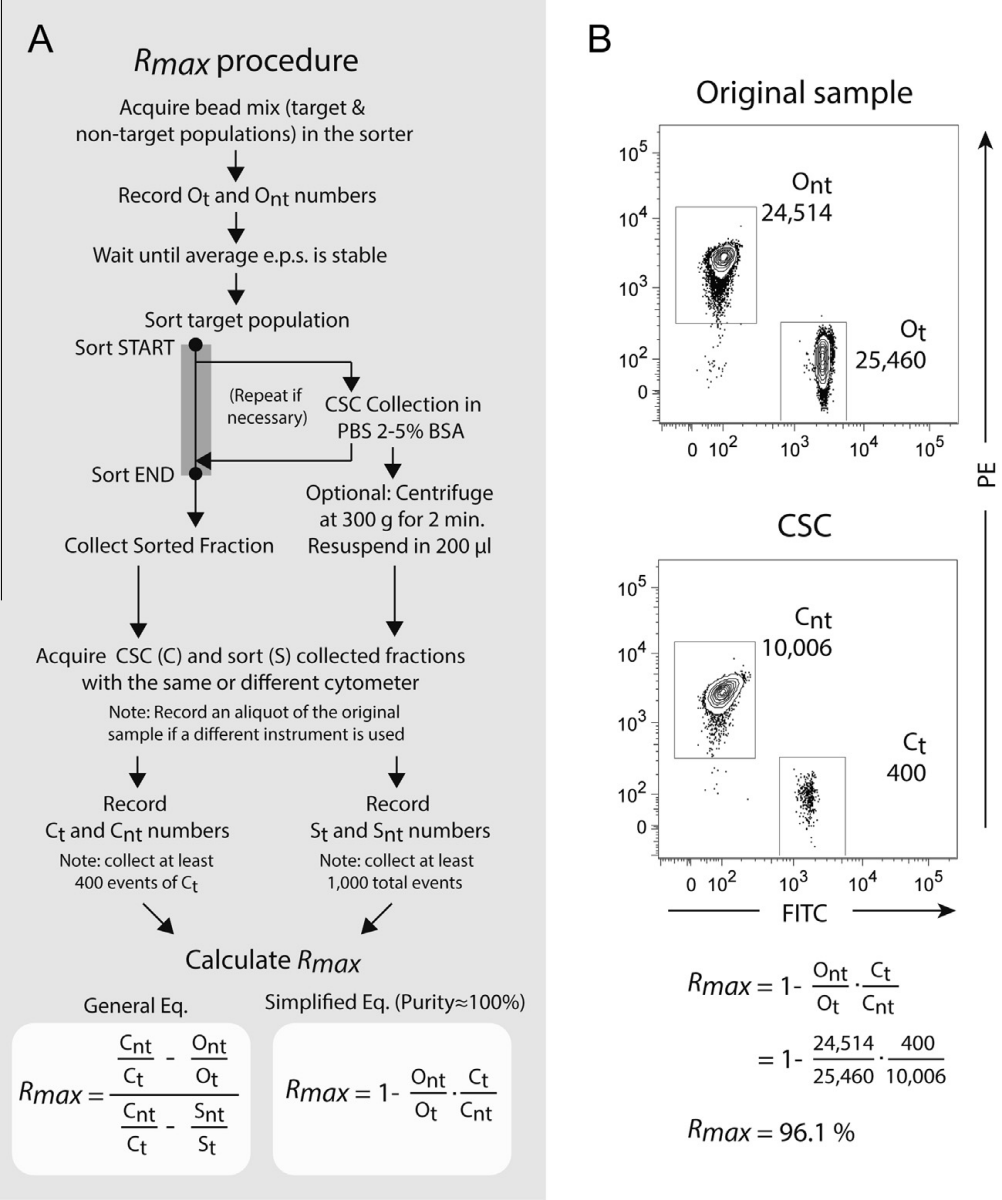
Summary of the general *Rmax* procedure used to evaluate sorter performance as a daily QC check before sort, during sort, or to assess instrument-specific drop-charge delay methods (A) simplified diagram of how to perform *Rmax*. (B) *Rmax* determination as a daily QC check with a FACSAria (70 μm nozzle) using an original mix of FITC (target, *O_t_*) and PE (non-target, *O_nt_*) CaliBRITE™ beads at an approximate 1:1 proportion, and sorting at 0-16-0 *Purify* mode. Gates should be drawn to include all positive events, being important to use positively-stained particles for both target and non-target populations. Center stream catch (CSC) was collected and target (*C_t_*) and non-target (*C_nt_*) events were recorded and replaced in Eq. (13).

Performing *Rmax* is simple and straightforward. Fig. 1A shows a simplified diagram of the *Rmax* procedure. With a perfectly functional and optimally calibrated sorter, *Rmax* estimates should match the expected theoretical values given by the P–B theory, driven exclusively by droplet generation frequencies, total event rate, and original target frequencies (*O_t_*). When sorting cells, however, it may be hard to define this theoretical value given cell arrival times may not follow Poisson statistics. Furthermore, it may not be feasible to maintain a low or even stable sample rate, affecting the overall efficiency of the sort and consequently impacting on expected Recoveries. To evaluate instrument performance in terms of Recovery it is then critical to first estimate *Rmax* under ideal conditions to eliminate factors that can contribute to deviations in Recovery outcome from expected theoretical values. This can be done by sorting ideal particles such as fully mono-dispersed beads at event rates well below the drop-drive frequency to ensure 100% efficiency. In these conditions, theoretical Recovery should be 100%, and any particle loss can be attributed solely on instrument factors, such as inaccuracies in drop-charge delay estimation, fluidic perturbations, particle-jet interference and/or issues with the sort electronics.

To illustrate how *Rmax* can be used to evaluate instrument performance, we sorted FITC^+^ (target) from a mix with PE^+^ (non-target) CaliBRITE™ beads at a frequency of 1:1 target to non-target ratio using a 0-16-0 *Purify* mode (Fig. 1B). In a FACSAria with a droplet-drive frequency of 90 kHz, the number of total events per second during sort was maintained below 1000 e.p.s. to ensure 100% efficiencies as displayed by FACSDiva software. The CSC was collected and analyzed in the same instrument. The number of target and non-target events in the original bead sample and CSC were recorded and used to calculate *Rmax* according to Eq. (13), which assumes Purity close to 100%. In this example in Fig. 1B, the calculated *Rmax* was 96.2% (a Supplementary spreadsheet is provided to help calculate the complete and simplified forms of *Rmax*). Deviations of *Rmax* from the expected value of 100% in these conditions can reflect inaccuracies in drop-charge delay determination, as well as other minor issues related to instrument fluidic instability or electronic failures such as imprecise drop charging. Since the drop-charge delay is suboptimal for particles of different sizes than the size of the particles used to determine the drop-charge delay [21], the deviation from the expected value may simply reflect the size differences between the CaliBRITE™ beads (6 μm) used to determine *Rmax* and the SPHERO™ Drop Delay Calibration Particles (7.2 μm) or the Flow-Check™ Fluorospheres (10 μm) used to determine the drop-charge delay in the FACSAria and MoFlo, respectively.

**Fig. 2:**
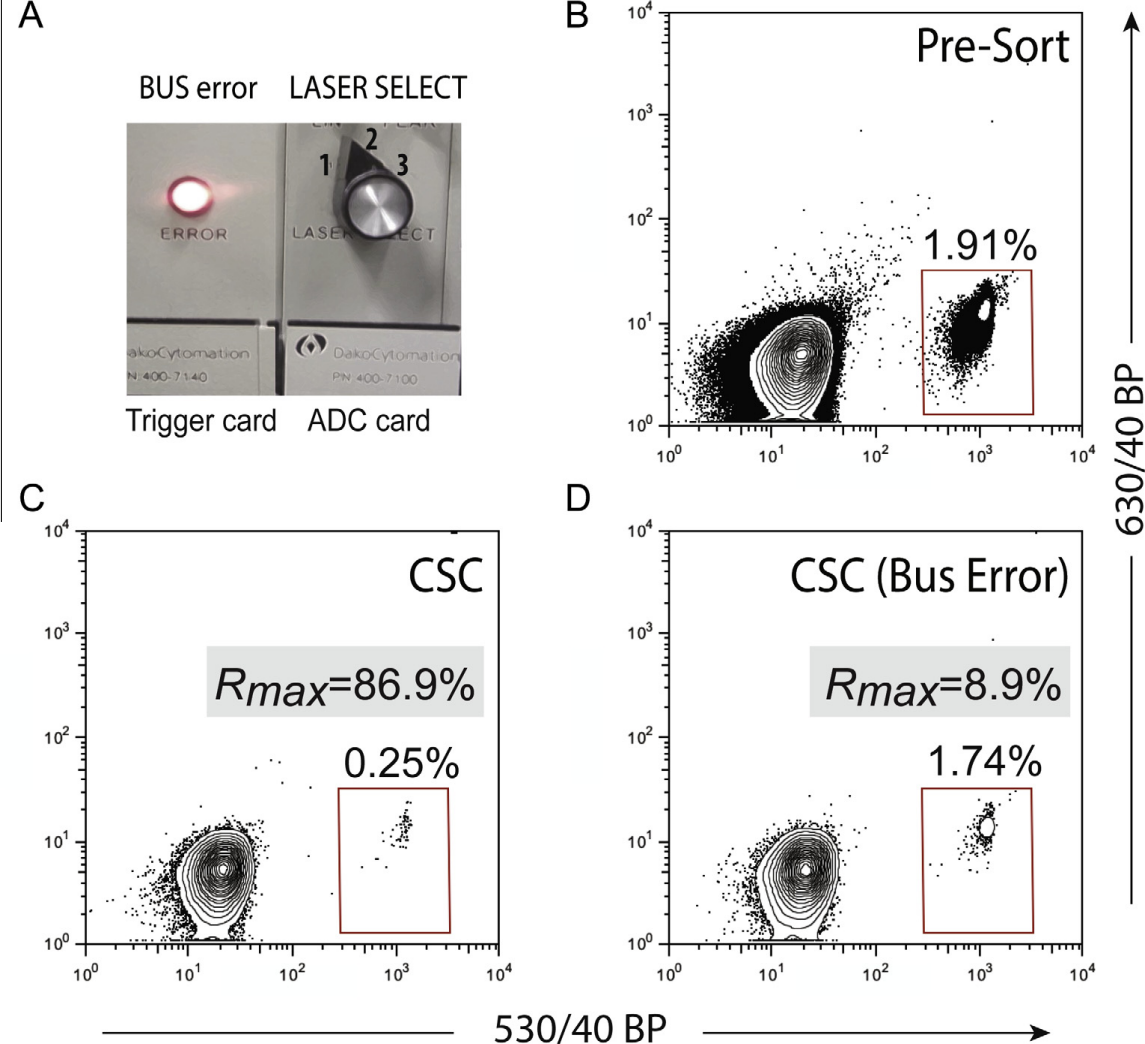
*Rmax* detects instrument-related sort failures. (A) Electronic bus errors imposed in a MoFlo by setting the ADC LASER SELECT knob in between laser delay positions. In a MoFlo with 100 μm nozzle (B) blank and FITC CaliBRITE™ Beads were mixed to give approximately 2% FITC bead concentration. A gate was set to sort all the FITC beads and (C) the CSC collected and re-analyzed. (D) CaliBRITE™ bead sorting was repeated under the imposed electronic error setting. Eq. (15) was used to calculate the *Rmax* values.

*Rmax* was also used to ascribe instrument failure in experiments in which an electronic error condition was imposed on the trigger card of the MoFlo electronics by setting one of the ADC LASER SELECT switches in between laser delay positions as illustrated in Fig. 2A. Sorting a sample with this forced error will result in the instrument sporadically ignoring some of the particles at the laser interrogation point resulting in such particles ending up in the waste. Using a MoFlo configured with a 100 μm nozzle, CaliBRITE™ FITC^+^ (target) beads were sorted from a mix with CaliBRITE™ blank (non-target) beads with a *Purify 1-drop* mode (Fig. 2B). The CSC was collected (Fig. 2C) and *Rmax* calculated upon analysis of the collected CSC, sort fraction and original samples. Singlet FITC^+^ bead sort was repeated under the same conditions as described above (Fig. 2D), except this time the trigger card electronic error was induced while sorting. The deleterious effect of this simulated electronic malfunction on target sort Recovery can be appreciated by a massive drop in the value of *Rmax* from the previous value of 87.15% to 9.05% under the hardware error condition.

### 3.3 Validating *Rmax* as a good estimate of instrument Recovery

**Fig. 3:**
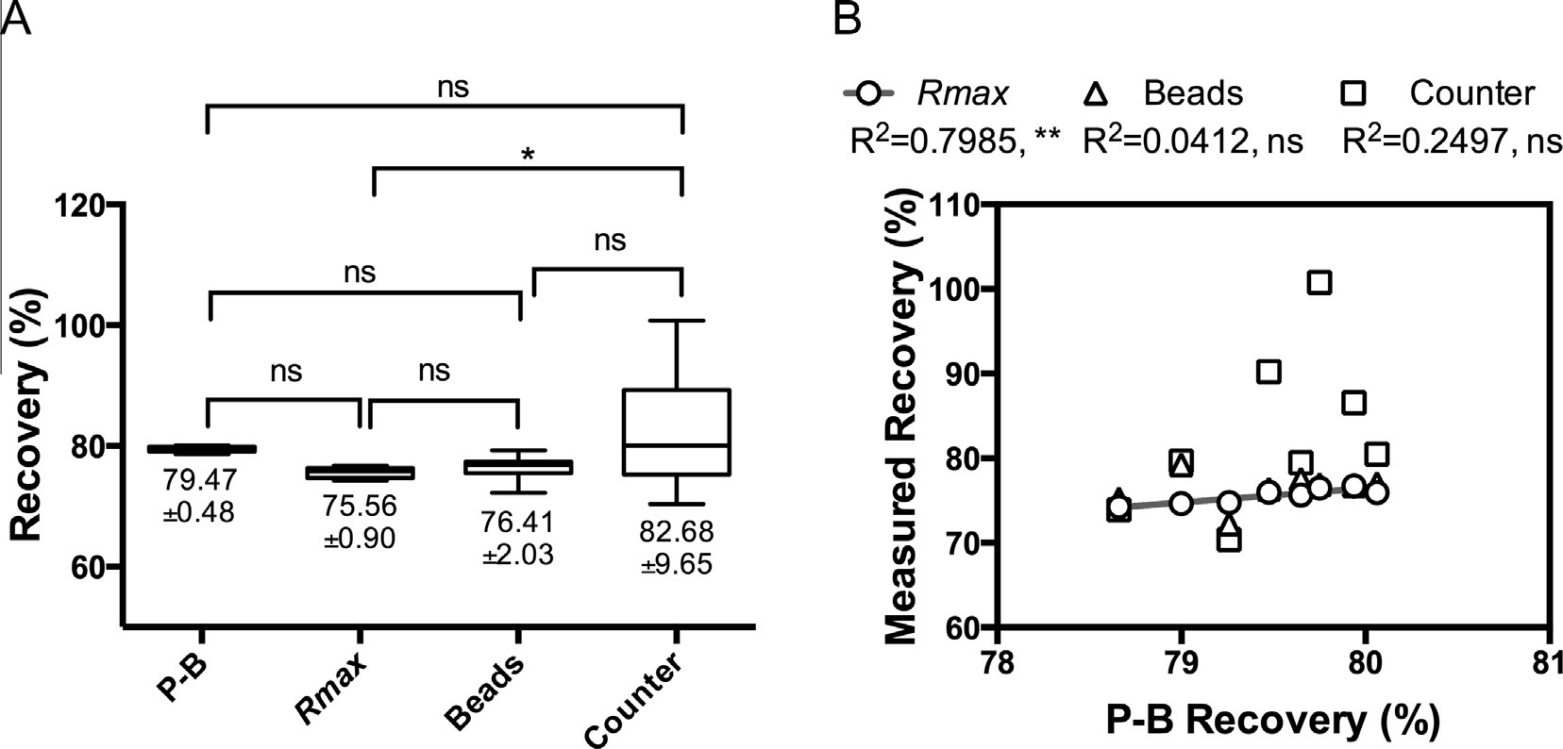
(A) Box and whisker plots for sort Recovery calculations based on Poisson and Binomial statistics, absolute counting methods and *Rmax* for replica *Single 1-drop* mode sorts of FITC^+^ CaliBRITE™ beads ran on a MoFlo Legacy cell sorter. Poisson–Binomial (P–B) Recovery was calculated as the number of sort decisions (*Sd* = 10^5^ FITC^+^ singlet CaliBRITE™ beads per sort) divided by the total number of target particles acquired for each sort (*O_t_*), estimated by adding the expected target contribution of drop and hard aborts estimated with Poisson and Binomial probability theory to the number of sorted target particles *St*. CountBright™ beads and slide-based BioRad TC-10 automatic counter were used to measure concentrations and absolute numbers of *S_t_* in the sort fractions. In both cases, sort recoveries were calculated by dividing the measure target particle counts in the sort fraction *S_t_* by the original number of target particles *O_t_* ran during each sort estimated as above from Poisson–Binomial (P–B) theory. *Rmax* was calculated based on Eq. (13). Numbers below each box represent mean Recovery values for each data set ± SD (*n* = 8). Boxes extend from 25th to 75th percentile values, lines in the middle of the boxes represents median values and whiskers represent minimum and maximum values of Recovery per set. (B) Correlation of Recovery replica values from *Rmax* and absolute counting-based methods with Poisson and Binomial derived Recovery data measured with Pearson product-moment correlation coefficient (*R*^2^). Linear regression between replica values of *Rmax* and P–B Recovery is also shown.

Evaluating the actual Recovery of a given sort typically relies on direct measurements of the absolute number of target particles in the sorted and original fractions. To validate *Rmax* as a reliable estimate of instrument Recovery, *Rmax* values were compared with traditional absolute count-based methods as well as with the theoretical maximum achievable Recovery to be expected out of sort modes aiming at high Purity. Values of this theoretical limit can be calculated based on Poisson probabilities of n-particle coincidence cases and the Binomial distribution defining the nature of the coincident particles. Fig. 3 compares *Rmax* to expected Recovery based on Poisson and Binomial theory (P–B) and traditional approaches relying on absolute counts of *S_t_*. Sequential replica sorts (*n* = 8) of 10^5^ singlet FITC^+^ beads out of a mix containing FITC^+^ and PE^+^ CaliBRITE™ beads, were performed on a MoFlo cell sorter at stable droplet generation frequency of 96.43 kHz and stable average sample speed of around 5000 e.p.s. A *Single 1-drop* sort mode was chosen to match the number of sort decisions reported by the electronics to the number of sort classified target particles (*Sd* = 10^5^). The number of droplet aborts reported by the instrument electronics upon completion of each sort was recorded, together with the average rate of total triggering events and the percentage of singlet FITC^+^ target particles out of the total triggering events as reported by Summit software. The number of original target particles (*O_t_*) ran by the instrument upon sort completion was calculated taking into account Poisson and Binomial probability-derived contributions of instrument-reported drop aborts and expected hard aborts to target particle numbers. Sort Recovery calculated under these sort conditions based on Poisson and Binomial estimations of *S_t_* yielded a mean value of 79.47 ± 0.48%. Since Recovery calculated this way is a direct function of the frequency of droplet generation, the average event rate, and frequency of target particles in the original sample (%*O_t_*), the limited variability observed confirms sort parameter consistency among the several sort replicas analyzed. Recovery values were similar among sort Recovery methods based on absolute counts of target particles in the sorted fractions (*S_t_*) with CountBright™ beads (76.41 ± 2.03%) showing a better reproducibility among data points than BioRad automatic counter (82.68 ± 9.65%). In the calculation of *Rmax*, the original pre-sort, sorted fraction and the CSC collected midway through the sort were analyzed in a flow cytometer. *Rmax* Eq. (13) was used, since Purity was near 100% in all cases. The calculated values of Recovery by *Rmax* were similar to the recoveries from absolute count-derived methods with a mean of 75.56 ± 0.90%. Both *Rmax* and bead-based Recovery values seem smaller than those calculated based on Poisson–Binomial theory, although their difference is not statistically significant. Additionally, the dispersion of *Rmax* values seems lower than that achieved by the absolute count-based methods, closer to the minimum variation shown by Poisson–Binomial derived data, suggesting a higher precision for this method when compared to traditional counting-based approaches. Fig. 3B shows the correlation between experimental Recovery methods and the expected Recovery based on Poisson–Binomial theory. Out of the three methods, only *Rmax* data shows a correlation with P–B Recovery values. This implies that the differences in calculated *Rmax* among sorts, although small, can be partially explained by variations in factors affecting P–B Recovery outcome, most likely changes in sample rate, since %*S_t_* and the frequency of droplet generation were constant for all the replica sorts. This further supports the precision and accuracy of the *Rmax* method, since the consistency in this metric among replicates is most likely affected by, and sensitive to, slight changes in P–B Recovery factors, and less to errors associated with the *Rmax* method itself.

### 3.4 Using *Rmax* to evaluate the effect of drop-charge delay estimations on sort Recovery

An accurate assessment of the drop-charge delay time is essential in order to achieve optimal sort performances. Sub-optimal assessments will lead to mismatches between jet charging and particle arrival times at the BOP, with a consequent reduction in target recoveries during the sort process. The Purity of the sort fraction however should not be compromised by small timing mismatches, unless the drop-charge delay is out by one or more drops when the chances of a target particle being sorted will be directly governed by *O_t_* frequencies.

**Fig. 4:**
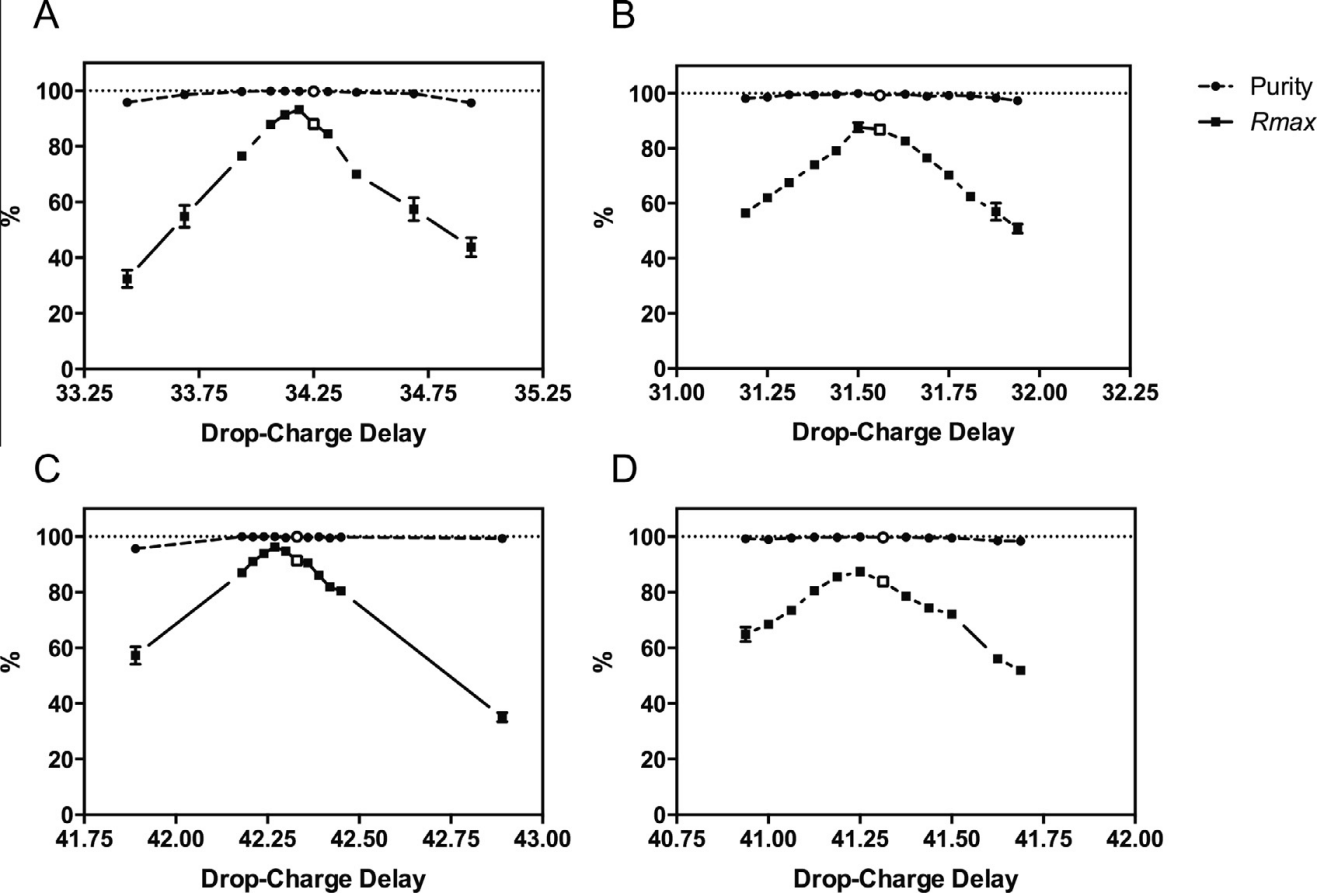
Purity and *Rmax* measured for several manually entered drop-charge delay values around Accudrop™ and Calibrator reported optimum drop-charge delay settings (Purity and Recovery results for this drop-charge delay point are represented in white). The tests were split between two sites. The Cell Imaging Unit, Instituto Gulbenkian de Ciência, MoFlo (A) and FACSAria 1 (C) were both configured with a 70 μm nozzle. The second site was the FCCF EMBL-Heidelberg with their MoFlo configured with a 70 μm nozzle (B) and a 100 μm nozzle (D). All sorted in *Purify 1-drop* mode in the MoFlo and 0-16-0 sort precision mode in the FACSAria.

We evaluated the dependency of Purity and Recovery on the accuracy of the drop-charge delay calculation in a MoFlo and FACSAria I cell sorters. Initially, optimum drop-charge delays were identified for the MoFlo, with the Calibrator device and for the FACSAria I we used the Accudrop system as recommended by the manufacturers. A mix containing a 1:10 ratio of FITC^+^ to blank CaliBRITE™ beads was acquired at a stable average sample rate of 10^4^ e.p.s. and sort target gate was defined around singlet FITC^+^ events. Triplicate sorts in *Purify 1-drop* mode were performed at the estimated optimal and neighboring drop-charge delays, spanning the extension of a drop in 1/16th of a drop steps. Fig. 4 shows the values of sort Purity and *Rmax* Recovery as functions of the drop-charge delay settings in the MoFlo (A, B and D) and the FACSAria (C) cell sorters. In both instruments Purity is maintained close to 100% over the entire range of drop-charge delays shown. Even towards the extremes of the drop-charge delay values, we could observe acceptable Purities greater than 95%. However, *Rmax* values show a strict dependency on drop-charge delay, with a rapid Recovery decline as drop-charge delays move away from optimum values. Surprisingly, the optimal drop-charge delay in terms of *Rmax* Recovery in both instruments seems to differ from the expected and initially defined optimal drop-charge delay, suggesting inaccuracies in both the FACSAria’s Accudrop™ and MoFlo’s Calibrator droplet inspection methods. Optimum drop-charge delays, defined as the timing for maximum *Rmax* (corresponding to minimum *C_t_* percentages; not shown), were out by 1/16 to 2/16th of a drop from the initially estimated optimal drop-charge delay settings in the FACSAria and MoFlo.

**Fig. 5:**
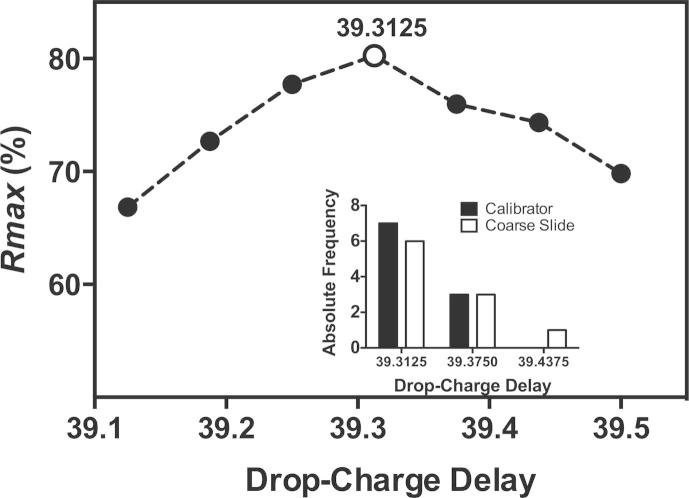
MoFlo equipped with a homemade Calibrator and 70 μm nozzle was run at 414 kPa (60 psi) and 96 kHz droplet generation frequency. Drop-charge delay was measured (*n* = 10) with the Calibrator and MoFlo’s coarse slide method. Drop-Charge delay was also evaluated by *Rmax* while sorting CaliBRITE™ beads mix in Single 1.0 mode at several drop-charge delay settings around the estimated optimal value of 39 + 5/16 (*n* = 7).

We further investigated the extent of the accuracy of our MoFlo’s calibrator device to identify the optimal drop-charge delay (Fig. 5). In order to prevent bias in value estimations, one of the authors (AR) identified the drop-charge delay settings providing minimal Flow-Check™ bead flashing at the center stream through inspection of real-time video camera images while blindingly scanning MoFlo drop delays with the instrument electronics control knob. A second author (APG) annotated the reported optimal drop-charge delays and randomly re-positioned the drop delay control knob back to suboptimal settings in between measurements. The instrument drop-charge delay was additionally estimated using Summit’s coarse drop delay procedure while sorting Flow-Check™ beads on slides [2]. Both the real-time CSC inspection and coarse slide methods showed variations in drop-charge delay estimation. Real-time center stream inspection provided the most accurate estimations of drop-charge delays with 7 out of 10 reporting a value of $39+\frac{5}{16}$, whereas 5 out of 10 coarse slide measurements reported the same value. *Rmax* was calculated for replica *Single 1-drop* sorts (*n* = 3) of singlet FITC^+^ CaliBRITE™ beads out of a mix containing around 1:1 ratio of PE^+^ and FITC^+^ beads. Several drop-charge delay settings were tested, including the above estimated $39+\frac{5}{16}$and surrounding drop-charge delays ($39+\frac{3}{16}$ ⩽ drop-charge delay ⩽ 39+$\frac{8}{16}$). The highest Recovery was achieved while sorting with a $39+\frac{5}{16}$ drop-charge delay setup (*Rmax* = 80%), whereas sorts performed with neighboring drop-charge delays showed a gradual drop in Recovery the farthest away from the optimal value, similar to results shown in Fig. 4.

## 4 Discussion

In *Enrich* or *Yield* mode sorting, the primary goal is to obtain as many cells of interest as possible, and therefore measuring Recovery to assess instrument performance is crucial. Yet, current methods to measure Recovery rely on counting the end-sort product, which is ultimately dependent on both instrument performance and sample loss, preventing researchers from understanding whether loss of Recovery is due to instrument or sample, or both. *Rmax* does not measure particle losses outside of the instrument, only particles that are “seen” by the instrument are considered, making *Rmax* ideal in instrument troubleshooting. For this same reason, *Rmax* cannot be used directly to troubleshoot sample preparation. Yet by assessing instrument performance using *Rmax*, any significant loss of cells compared to the *Rmax* estimation can be attributed to other factors related to sample preparation or processing. Standard methods of determining Recovery also require an accurate counting of particles in the pre- and post-sort compartments. However, large errors are invariably introduced due to significant inaccuracies in traditional counting methods. Because the *Rmax* method relies on the ratios of target to non-target populations rather than the absolute values in the pre-sort sample, the post-sort sample and the CSC, the error in counting is greatly reduced.

When sorting in *Purify* or *Single-cell* modes, Recovery is still the primary metric to assess performance of a cell sorter, since factors affecting Purity will compromise Recovery, but issues compromising Recovery may not necessarily affect Purity. Measuring Purity may still be required in these sort modes, to assess the quality of the overall sort, but is not useful as a metric of instrument performance.

*Rmax* is quick and simple and can be performed at the start of each instrument setup by the operator. There is no need for added costly instrument modifications or for specially designed tracking particles. In the hands of a cytometry professional, our method could be used to dissociate issues of instrument malfunction and instrument setup from those related with poor sample handling before or after the sort.

### 4.1 Using *Rmax* as a quality control tool to assess instrument performance

When setting up the instrument for sort, *Rmax* can be used to evaluate how well the sorter is performing. By sorting a mix of target and non-target mono-dispersed beads that closely follow a Poisson distribution in terms of laser or BOP arrival times, *Rmax* can be measured and compared to the “ideal” Recovery in the same conditions. With a fully optimized and calibrated instrument, Recovery should be close to 100% as long as the “ideal” sample is sorted under conditions in which no sort-decision aborts take place, i.e., close to 100% efficiency of sort decisions. This can be achieved by running the bead mix at a total event rate well below the drop-drive frequency. The theoretical limits could be calculated using Poisson and Binomial probabilities as shown in the Results section, but are dependent on the frequencies of the target population compared to the total number of triggering particles in the sample. In practice, it is easier to simply adjust the flow-rate while sorting the “ideal” sample until the efficiency of the sort decisions given by the instrument is approximately 100%. In our case, sorting a 50% target frequency population in a 90 kHz drop-drive frequency (90,000 drops/s) we ran the sample at an average rate of 900–1000 e.p.s. during the sort. Though any original target frequencies can be used to measure *Rmax*, we found 50% target frequency to be the most adequate proportion. At lower, or much lower original target frequencies, chances of finding a target particle in a given volume of the CSC after sort becomes less likely. If insufficient CSC is collected, the number of target particles counted will be low, reducing the precision of *Rmax* estimates and therefore decreasing the sensitivity of the method. At higher or much higher original target frequencies, according to Fig. S1 in Supplementary materials, the estimate of *Rmax* calculated using the simplified expression (Eq. (13)) that relies in collecting only the CSC, will be significantly underestimated unless Purity is maintained above 98%, which may not be possible. At these high target frequencies, the accuracy of *Rmax* estimates is compromised. This can be overcome by measuring Purity and then using the full *Rmax* description given by Eq. (11), but will have the inconveniency of also having to measure the sorted sample. Even at 50% original target frequencies and capturing only the CSC, depending on the amount of measurements to be made and total sample rate while sorting, the CSC may be extremely diluted. In these cases we introduced a centrifugation step after collecting the CSC to spin-down the particles. This does not affect precision or accuracy because *Rmax* is dependent on the target:non-target ratio. Any particle loss by, for instance, sticking to the tube walls, will be proportionally distributed between target and non-target particles. It must be noted, however, that although measuring the ratios eliminates most of the experimental variation, the precision of the actual target and non-target values in each compartment – *O_t_*, *O_nt_*, *C_t_*, and *C_nt_* – is still dependent on Poisson counting. Therefore, a low count in any of these values will decrease the precision of *Rmax* estimation.

*Rmax* can also capture changes in sorter performance independent of inaccurate drop-charge delay determinations. If the instrument has a suboptimal flow-cell design or fluidics that affect the Poisson arrival times at the BOP, then maximum Recovery will be lower than the theoretical expectation of 100%. Yet, just as when performing QC on instrument sensitivity, *Rmax* can be used on a daily basis to monitor decreases in Recovery that then can be attributed to suboptimal drop-charge delay determinations, fluidic instabilities below the nozzle, or electronic errors. An actual baseline can be determined using the same approach as described in Fig. 4, by measuring *Rmax* at different drop-charge delay times around the measured delay obtained with the drop-charge delay method implemented in the instrument. The maximum *Rmax* estimate will be the baseline target value, which can be monitored for changes on a daily basis before each sort. Using *Rmax* as a performance check before sort can be particularly useful in single-cell sorting experiments. In particular, when sorting for single-cell genome or transcriptome studies, where a highly efficient particle deposition is crucial due to the typical high cost of reagents and resources involved in these experiments.

### 4.2 Using *Rmax* to assess instrument Recovery during cell sorting

In most cases, *Rmax* can be used to assess instrument Recovery during a sort experiment, taking advantage of the cell populations being sorted. To assure that *Rmax* determinations at different time points during the sort are comparable, the average event rate must be similar. Furthermore, the two cell populations defined as target and non-target must be of the same type, i.e., have similar size or adherence properties to eliminate any bias in *Rmax* calculations. For instance, target and non-target particles of different sizes may sediment at different rates and/or have different adherence properties, and therefore attach differentially to the tubing of the instrument or to the sample and collection tubes. This would violate the assumption used to derive Eq. (11) that the target:non-target ratio must remain identical while sorting a fraction *α* of the original sample. If the ratios remain identical while sorting a fraction *α* of the total sample but change significantly throughout the entire sort experiment – whether by differential sedimentation, adherence or even cell death –they will still have an impact on *Rmax* measured at different stages of the sort. Even taking into account the new target:non-target ratios used to find *Rmax* at each measured time-point, the actual Recovery is affected by changes in the target frequencies. Care must therefore be taken in interpreting these changes. Values of *Rmax* are only comparable under similar conditions, i.e., if the system is fairly stable in terms of total number of e.p.s and frequency of target particles relative to the total triggering population.

Measuring *Rmax* can be especially important during a rare cell sort. However, it is exactly under these conditions that the method is less sensitive unless a large volume of CSC is collected to count enough target particles. This limitation can be overcome as long as there are other cell populations in the sample of the same type that can be used as target and non-target populations. The most frequent population can be defined as the target population and sorted to a different tube, whereas the second most frequent population may serve as non-target. This will imply sorting an extra population with the sample, though sorting more than one population simultaneously will not impact *Rmax* given that a sort decision is made on every interrogated particle irrespective of whether the decision to sort is made or not. In contrast, adding a *new* population to the sample, such as bead particles to monitor *Rmax*, or simply by lowering the threshold, will have a negative impact on Recovery (and therefore *Rmax*). The extra population in the sample will decrease the frequency of target cells with respect to the total number of particles, and thus increase the probability of aborting decisions to sort target cells.

### 4.3 Using *Rmax* to evaluate instrument-specific drop-charge delay methods

When no fluidic instabilities are visible downstream of the nozzle while sorting, *Rmax* can be regarded as a measure of how well the instrument is charging the right drop at the right time. Therefore it is ideal to evaluate drop-charge determination methods supplied with the instrument. All methods are fallible and may have more or less precision, depending on the type of method and how well it is calibrated. For instance, the methods used in the present work to measure the drop-charge delay are based on the visual inspection of fluorescent beads being deflected correctly as one sets the optimal timing. Yet, suboptimal camera sensitivity or brightness and contrast settings, for instance, may reduce the accuracy of these methods. By measuring *Rmax* at different drop-charge time delays around the measured drop-charge delay determined by the manufacturer’s method (Figs. 4 and 5) it is possible to confirm the optimal charge delay. In our results, we observed occasional discrepancies between the optimal drop-charge delay found using *Rmax* and the delay determined by the method already implemented in the instrument (Fig. 4). Yet as previously reported [21] different size particles have different arrival times and therefore different optimal drop-charge delays. Since the beads used to measure *Rmax* and the drop-charge delay were of different sizes, the observed discrepancies can be simply a reflection of these differences. We could not find beads of the same size suitable both for the drop-charge delay methods of the instruments and *Rmax*. Still, this will require further investigation to understand the effects of size and its impact on Recovery, and the *Rmax* method is the suitable tool for that.

## 5 Conclusion

The entire *Rmax* method is straightforward. The target and non-target of the original sample are already displayed during a sort. Quick CSC collection, spin down and analysis on a separate flow cytometer will determine the *Rmax* value during a sort, leading to good estimates of sorting time and sort product number. *Rmax* can be used as QC tool for sorters, by assessing the maximum Recovery of the instrument using an “ideal” sample in “ideal” conditions, and can be particularly useful in single-cell sorting experiments. It can be used during a sort experiment to monitor Recovery throughout the sort by comparing the *Rmax* estimates with those obtained in the beginning of the sort. This is especially important for rare cell sorts where optimal Recovery is mandatory, and without compromising the sorted product. Finally we can use the method to directly compare instruments and their drop-charge delay determination methods, which is particularly useful when assessing new instrumentation.
