# Supplementary material for: Rmax: A systematic approach to evaluate instrument sort performance using center stream catch
Source: Methods. 2015 Jul 1;82:64–73. doi: 10.1016/j.ymeth.2015.02.017 (PMC4503806; doi:10.1016/j.ymeth.2015.02.017)
Supplement: Supplementary data 1 [file mmc1.docx]

# Exploring conditions in which simplified Rmax equations can be used

It is important to evaluate the conditions in which Eq.14 and 15 present the minimum acceptable differences relative to the full description of *Rmax* given by Eq.12. These limits will allow using the simplified definitions of *Rmax* to measure the maximum Recovery of the sorting instrument.

The absolute difference between the complete description of *Rmax* expressed in percentages (Eq.12) and the simplified equation when Purity is close to maximum (Eq.14) has the following expression:

g$Rmax-{Rmax}_{P\to1}=\frac{{\%C}_{t}-{\%O}_{t}}{{\%C}_{t}-{\%S}_{t}}\cdot\frac{{\%C}_{t}}{{\%O}_{t}}\cdot\frac{{100-\%S}_{t}}{{100-\%C}_{t}}$ (S1)

**Figure S1.** Contour plots represent the absolute difference between the complete *Rmax* description and the simplified *Rmax* equation for Purity close to maximum (Eq. S1) as a function of several starting target to non-target particle ratios (T:NT), Purity (%*S_t_*) and target particle loss (%*C_t_*). Purity and target loss ranges were constrained by the original target frequency (0 ≤ %*C_t_* ≤ %*O_t_*; %*O_t_* ≤ %*S_t_* ≤ 100), and only these plausible ranges are represented. Contour lines represent four differences 1% (light yellow), 2%, 5%, and 10% (red).

Figure S1 shows three-dimensional contour graphs of *Rmax* difference in Eq.S1 as a function of Purity (%*S_t_*) and target particle loss (%*C_t_*) for several ratios of original target to non-target particles. Drawing a vertical line across the contour plots in Figure S1 reveals the minimum Purity needed so that the difference between both estimates of *Rmax* is equal or less than the value represented by the respective contour line. For instance, with initial target frequencies of 50% or less, as long as Purity is above 96%, the difference between both estimates is under 1%, which is a good approximation. On a more conservative side, Purities of 93% will guarantee *Rmax* estimates less than 2% away from the full estimate, as long as initial target frequencies are 50% or less.

For Purity sorts with very low original target frequencies (Eq.15), a further simplification of *Rmax* can be made. The absolute difference between this estimate and the complete *Rmax* equation expressed in percentages (Eq.12) is given by:

$Rmax-{Rmax}_{p\to1,{\%O}_{t}\to0}=\frac{{\%C}_{t}-{\%O}_{t}}{{\%C}_{t}-{\%S}_{t}}\frac{{\%C}_{t}}{{\%O}_{t}}$ (S2)

This difference, calculated for each sorting scenario with Purities above 50% (Figure S2), can also be used to evaluate the best conditions in which the approximation is reasonable. Again, by drawing a vertical line, it is possible to find the maximum initial target frequency at which this line still intercepts the desired contour line, corresponding to the maximum %*O_t_* that will produce a difference in estimates equal or lower to the respective contour line. Figure S2 shows that, for Purities of 90% or higher, the approximation will be less than 1% away from the full estimate of *Rmax* as long as the initial target frequency is less than 4%.

**Figure S2.** Absolute differences in percentage between the complete description of *Rmax* and the simplified *Rmax* estimated for high purity sorts with low original target frequencies (Eq.S2). Each plot represents the differences in percentages for a given sort Purity value (%*S_t_*), as a function of initial target frequency (%*O_t_*) and target particle loss (%*C_t_*). White area in plots represents a range of pair-wise values for %*O_t_* and %C_t_ that are not physically plausible, since %*O_t_* ≥ %*C_t_* when the instrument is set to sort target particles. Contour lines represent three differences: 1%, 2%, and 5%.
