# Supplementary material for: Rmax: A systematic approach to evaluate instrument sort performance using center stream catch
Source: Methods. 2015 Jul 1;82:64–73. doi: 10.1016/j.ymeth.2015.02.017 (PMC4503806; doi:10.1016/j.ymeth.2015.02.017)
Supplement: Supplementary data 3 [file mmc3.docx]

Rmax Calculator in: Cytometry Toolkit for iOS and Android

**Google Play:**

<https://play.google.com/store/apps/details?id=uk.co.riddell.cytoTool&feature=search_result#?t=W251bGwsMSwyLDEsInVrLmNvLnJpZGRlbGwuY3l0b1Rvb2wiXQ>..

**iOS**

<https://itunes.apple.com/gb/app/cytometry-toolkit/id564167182?mt=8>

**Author:**

Andy Riddell

**Contact:**

Andy Riddell

Flow Cytometry Core Facility Manager

Wellcome Trust-MRC Stem Cell Institute,

Centre for Stem Cell Research

University of Cambridge

Tennis Court Road

Cambridge

CB2 1QR

UK

Tel: +44 (0)1223 760221
